# 8,888 Newborns Over One Year: Evaluation of a Universal Neonatal Hearing Screening Program

**DOI:** 10.1055/a-2804-5197

**Published:** 2026-09-29

**Authors:** Laura Franco Chiriboga, Karolina Pessote Sideri, Jonathan Grassi, Elaine Soares Monteiro Pinto, Luis Miguel Chiriboga Arteta

**Affiliations:** 1 PhD in Health, Interdisciplinarity, and Rehabilitation Universal Neonatal Hearing Screening Program, Hospital Maternidade de Campinas, Campinas, São Paulo, Brazil; 2 PhD in Child and Adolescent Health Universal Neonatal Hearing Screening Program, Hospital Maternidade de Campinas, Campinas, São Paulo, Brazil; 3 PhD in Public Health Federal University of Espírito Santo - Vitória/Espírito Santo – Brazil; 4 PhD in Communication Disorders Universal Neonatal Hearing Screening Program, Hospital Maternidade de Campinas, Campinas, São Paulo, Brazil; 5 Doctor Otorhinolaryngologist, Universal Neonatal Hearing Screening Program, Hospital Maternidade de Campinas, Campinas, São Paulo, Brazil

**Keywords:** hearing, hearing loss, neonatal screening

## Abstract

**Introduction:**

Universal Newborn Hearing Screening is essential for early detection of hearing loss, enabling prompt interventions and improving child development.

**Objective:**

To analyze the outcomes of a universal neonatal hearing screening program.

**Methods:**

Retrospective descriptive study analyzing the results of neonatal hearing screening. The inclusion criterion was birth at or transfer to the hospital between January and December 2023. The research was approved by the Research Ethics Committee, under number 5.227.720/2022.

**Results:**

8,828 (99.32% of total sample) underwent screening, with 7,846 having no risk factors for hearing loss and 982 identified with risk factors for hearing loss. Among the 60 newborns who were not screened, 7 (0.08%) were transferred to other hospitals before undergoing the universal neonatal hearing screening, 50 (0.56%) passed away, and 3 (0.03%) were lost to follow-up and did not undergo the screening. For the TEOAE and aABR tests, the variables sex, type of healthcare service, and birth weight had a significant effect on increasing the likelihood of test failures.

**Conclusion:**

The outcomes of one year of the UNHS service were assessed, revealing a coverage rate of 99.96%. The service meets the quality indicators recommended by the guidelines, except for the percentage of referrals for diagnosis. Although challenges remain and new goals are yet to be achieved, the importance of conducting further research is recognized to monitor the auditory development of infants throughout life, especially those with risk factors for hearing loss.

## Introduction

The demographic and epidemiological transition in countries regardless of income level has reshaped risk profiles for chronic conditions, including childhood hearing loss (HL). Early intervention and treatment remain the most effective strategies to mitigate its impact, promoting speech, language, academic, and social development. 1 2 3 Developed nations have long implemented Universal Neonatal Hearing Screening (UNHS), providing valuable insights into early HL management. 2 3 4 5 6

The Joint Committee on Infant Hearing (JCIH) 7 endorses UNHS for all newborns, irrespective of risk factors. In Brazil, the Ministry of Health offers guidance for implementing UNHS services through its Neonatal Hearing Screening Care Guidelines. 8

Despite increased neonatal hearing screening coverage in Brazil, national rates remain low, 9 10 revealing gaps in reaching all newborns and meeting recommendations. This article aims to describe the outcomes of a Brazilian UNHS program.

## Methods

The study adhered to STROBE guidelines11 to enhance the reporting of observational studies.

### Ethical Aspects

The research was approved by the Research Ethics Committee, under number 5.227.720/2022. As this was a retrospective study involving medical records review, it was exempt from the requirement to obtain informed consent.

### Study Design

This study analyzed medical records from the UNHS program at a hospital in Campinas, Brazil, from January to December 2023. The sample included 8,888 neonates born at or transferred to the hospital during this period. Inclusion criteria were newborns delivered or transferred within this timeframe, while exclusions included those who missed UNHS due to transfer, evasion, or death. The final sample comprised 8,828 newborns, with 7 transferred, 50 deceased, and 3 lost to follow-up.

### Procedures

The hospital's UNHS program follows the Ministry of Health guidelines 8 and the Joint Committee on Infant Hearing. 7 UNHS is conducted within 24 to 48 hours of life, except for neonates in the ICU. It includes two stages: the test stage (before discharge) and the retest stage (after discharge), as shown in Figure 1 .

**Fig. 1:**
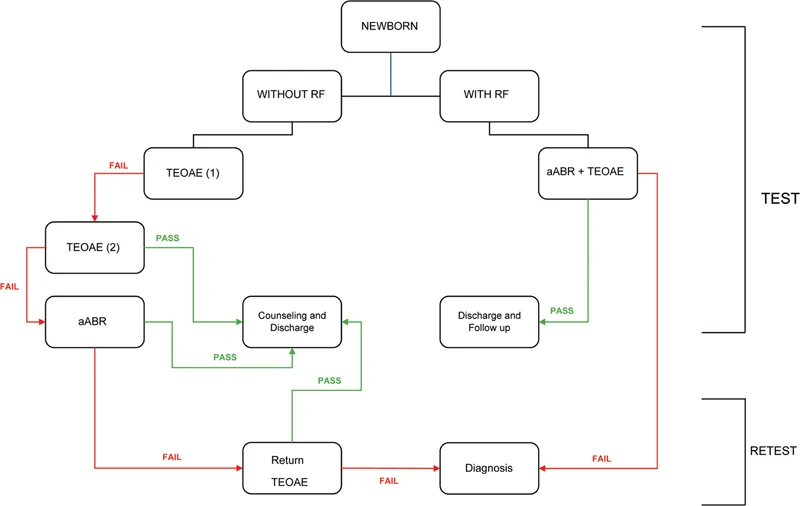
Flowchart of Universal Neonatal Hearing Screening.

For newborns without hearing loss risk factors, Transient Evoked Otoacoustic Emissions (TEOAE) is used. For those with risk factors (RF, as defined by the Ministry of Health and JCIH guidelines), Automatic Auditory Brainstem Response (aABR) is performed, with TEOAE as a complementary test. The "pass criterion" is based on the aABR result.

If a newborn without risk factors fails the TEOAE, the test is repeated within 8 hours. If the failure persists, aABR is performed to reduce false positives. If the result remains a failure, a follow-up is scheduled within 15 days, and further audiological diagnosis is provided if necessary.

Newborns with risk factors who pass the aABR are referred for audiological follow-up at six months or corrected age, using behavioral assessment and visual reinforcement audiometry. Those who fail the aABR are directly referred to audiological diagnosis.

All exams were conducted by trained audiologists using equipment that provides automatic responses for TEOAE and aABR tests, indicating either "pass" or "fail." For TEOAE, two devices were used: MADSEN Accuscreen – Otometrics and Sentiero – Path Medicals. Both use click stimuli, with automatic result analysis. They differ in intensity (70-84 dB SPL for Accuscreen, 85 dB SPL for Sentiero) and frequency range (1.5-4.5 kHz for Accuscreen, 1-4 kHz for Sentiero).

For aABR testing, three devices were used: MADSEN Accuscreen – Otometrics, Eclipse – Interacoustics, and Sentiero – Path Medicals. All operated in automatic mode at 35 decibels normalized hearing level (dBnHL), using statistical tests for result analysis. The Accuscreen and Eclipse use click stimuli, while Sentiero uses chirp stimuli. Eclipse and Sentiero are also used for audiological diagnosis.

### Statistical Analysis

Study data were analyzed using Microsoft Excel for sample characterization, recording, and interpretation of findings, without influencing the existing variables.

The significance level was set at 5% (p ≤ 0.05). Results were interpreted based on both statistical significance and effect size, as the p-value can be influenced by sample size, while effect size assesses the practical impact. 12 13 SPSS Statistics software (version 27.0; IBM Corp., Armonk, NY, USA) was used for all analyses, following the theoretical framework detailed by Field.14 14

To evaluate the influence of risk factors for hearing loss, sex, type of delivery, healthcare service, and birth weight on TEOAE test failures, generalized linear univariate and multivariate logistic models (GzLM) with a logit link function were developed for each ear. Multicollinearity indices were within acceptable limits (VIF ≤ 1.145; tolerance ≥ 0.873; mean VIF = 1.079). Due to missing data, univariate models for sex, type of delivery, and birth weight included n = 8,827, while the final multivariate model included n = 8,825. For other models, the total sample size (n = 8,828) was used.

When variable interactions were identified in the multivariate model, post hoc analyses with Bonferroni correction were performed to clarify these interactions. GzLM were also constructed to evaluate the influence of sex, type of delivery, healthcare service, and birth weight on aABR failures, using the same parameters and procedures as for TEOAE testing.

The models showed acceptable multicollinearity indices (VIF ≤ 1.037, tolerance index ≥ 0.964, mean VIF = 1.025). Due to missing data, univariate models for sex and type of delivery included n = 980, while the final multivariate model included n = 979. The full sample (n = 981) was used for other models.

OR values were interpreted using Chen et al.'s 15 classification: Small (0.30–0.60 or 1.68–3.46), Medium (0.15–0.29 or 3.47–6.70), and Large (≤ 0.14 or ≥ 6.71).

## Results

Of the 8,888 newborns born or transferred to the hospital, 8,828 (99.32%) underwent screening. Among them, 7,846 (88.88%) had no risk factors, and 982 (11.12%) had risk factors for hearing loss. Of the 60 not screened, 7 (0.08%) were transferred, 50 (0.56%) passed away, and 3 (0.03%) were lost to follow-up. The coverage rate was 99.96%.

Table 1 shows the sample distribution by sex, type of delivery, healthcare service, and birth weight. One intersex individual was excluded, and one case with missing data for delivery type and birth weight reduced the sample to 8,827 for these variables.

**Table 1:** Sample characterization by sex, type of delivery, type of healthcare service, and birth weight

	Category	n (%)
Sex	Female	4304 (48.76)
	Male	4523 (51.24)
Type of delivery	Vaginal	3065 (34.72)
	Cesarean	5762 (65.28)
Health Service	Public	5225 (59.19)
	Private	3603 (40.81)
Birth weight (g)	Mean (SD)	3,135.75 (546,96)
	Median (Min–Max)	4,990.00 (575-5565)

Abbreviations: g, grams; Max, maximum; Min, minimum; SD, standard deviation.

### Analysis of Results for Newborns Without Risk Factors

Figure 2 shows the results for newborns without risk factors.

**Fig. 2:**
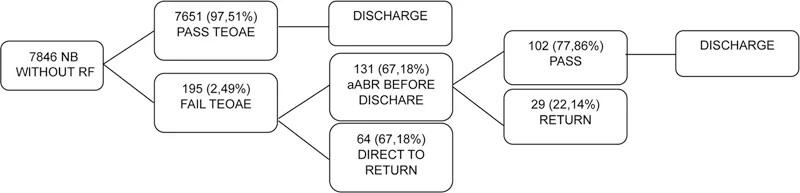
Description of the neonatal hearing screening results for newborns without risk factors.

A total of 93 newborns (1.18% of newborns without risk factors) required retesting after discharge—reduced from 195 by using the combined methodology (aABR before discharge). Of these, 85 attended, while 8 (8.60%) were lost to follow-up. In the retest, 80 (86.02%) passed the TEOAE, and 5 (5.38%) failed and were diagnosed with hearing loss ( Fig. 3 ).

**Fig. 3:**
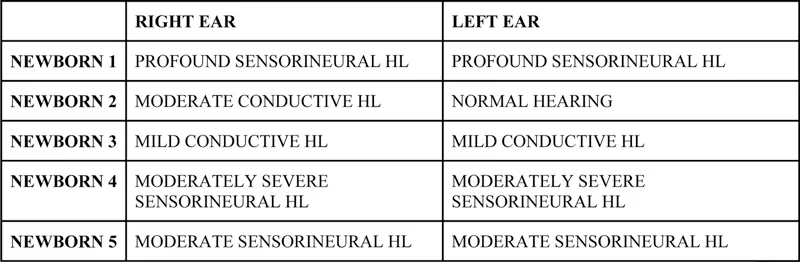
Description of hearing loss diagnosed in newborns without risk factors.

All diagnosed newborns were counseled and referred for otolaryngological evaluation and management.

### Analysis of Results for Newborns with Risk Factors

Figure 4 shows the results for newborns with risk factors.

**Fig. 4:**
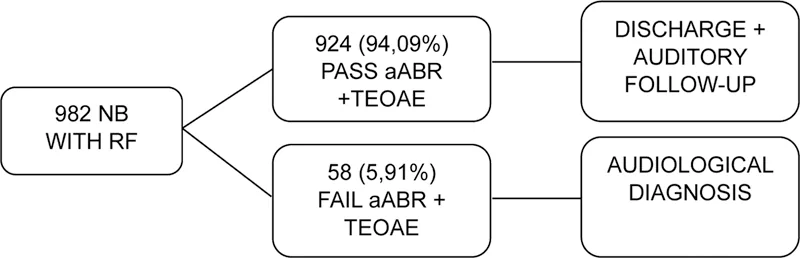
Description of the neonatal hearing screening results for newborns with risk factors.

In the audiological diagnosis post-discharge, 5 newborns (8.62%) were lost to follow-up, 40 (68.97%) had normal hearing and were referred for follow-up, 1 (1.72%) passed away, 1 (1.72%) was transferred, and 11 (18.97%) were diagnosed with hearing loss ( Fig. 5 ).

**Fig. 5:**
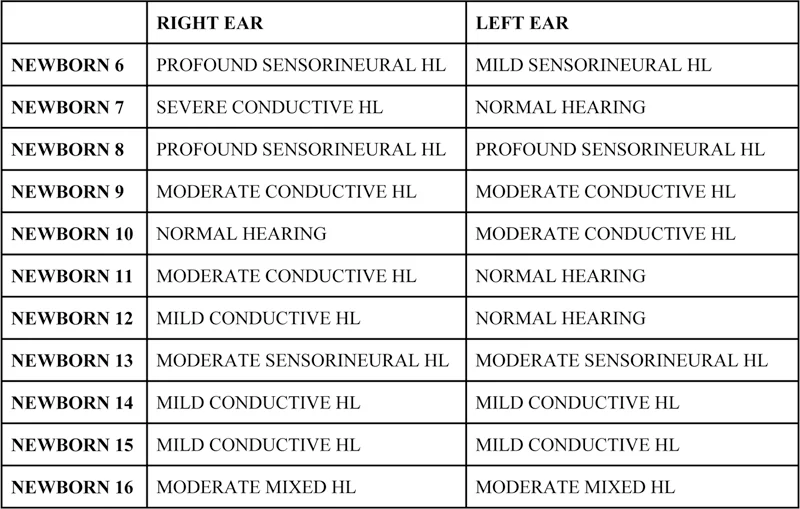
Description of hearing loss diagnosed in newborns with risk factors.

All diagnosed newborns were counseled and referred for otolaryngological evaluation and management. No cases of auditory neuropathy spectrum disorder were identified.

### Analysis of the Relationships and Influences of Variables on the Results for each Ear

Table 2 shows the distribution of TEOAE and aABR test results by ear.

**Table 2:** Characterization of the sample regarding the results of TEOAE and aABR testing by ear

Procedure		RE		LE	
		Pass	Fail	Pass	Fail
**TEOAE**	n (%)	8,656 (98.05)	172 (1.95)	8609 (97.52)	219 (2.48)
**aABR**	n (%)	932 (95.01)	49 (4.99)	937 (95.51)	44 (4.49)

Abbreviations: aABR, Automatic Auditory Brainstem Response; LE, left ear; RE, right ear; TEOAE, Transient Evoked Otoacoustic Emissions.

Tables 3 and 4 present the characterization of the sample regarding the presence of risk factors for auditory pathology, sex, type of delivery, type of healthcare service and birth weight, according to the results of the TEOAE and aABR screening.

**Table 3:** Occurrence of "Failure" results in TEOAE and aABR screenings according to the ear and the presence of risk factors for auditory pathology, sex, type of delivery and healthcare service

		TEOAE		aABR	
		RE	LE	RE	LE
		n (%)	n (%)	n (%)	n (%)
**RF**	No	116 (1.48)	164 (2.09)	–	–
	Yes	56 (5.70)	55 (5.60)	–	–
**Sex**	Female	59 (1.37)	73 (1.70)	16 (3.59)	14 (3.14)
	Male	113 (2.50)	146 (3.23)	33 (6.18)	30 (5.62)
**Type of delivery**	Vaginal	53 (1.73)	69 (2.25)	11 (4.03)	11 (4.03)
	Cesarean	119 (2.07)	150 (2.60)	38 (5.37)	33 (4.67)
**Health Service**	Public	89 (1.70)	119 (2.28)	22 (3.55)	22 (3.55)
	Private	83 (2.30)	100 (2.78)	27 (7.48)	22 (6.09)

Abbreviations: aABR, Automatic Auditory Brainstem Response; LE, left ear; RE, right ear; RF, risk factors; TEOAE, Transient Evoked Otoacoustic Emissions.

**Table 4:** Characterization of birth weight according to the ear and the results of the TEOAE and aABR screenings

Ear	Weight (g)	TEOAE		aABR	
		Pass	Fail	Pass	Fail
**RE**	**Mean (SD)**	3,139.96 (540.26)	2,922.37 (790.60)	2,625.52 (837.24)	2,415.92 (872.25)
	**Median (Min–Max)**	3.185 (575–5075)	3,040 (725–5,565)	2,800 (575–4,990)	2,585 (725–3,960)
**LE**	**Mean (SD)**	3,139.9 (540.66)	2,991.65 (742.08)	2,625.68 (834.36)	2,388.75 (930.30)
	**Median (Min–Max)**	3,185 (575–5,075)	3,070 (725–5,565)	2,800 (575–4,990)	2,435 (725–3,960)

Abbreviations: aABR, Automatic Auditory Brainstem Response; g, grams; LE, left ear; Max, maximum; Min, minimum; RE, right ear; SD, standard deviation; TEOAE, Transient Evoked Otoacoustic Emissions.

Table 5 presents the analysis of RF presence, sex, delivery type, healthcare service, and birth weight on TEOAE failures using univariate and multivariate logistic GzLM for each ear.

**Table 5:** Odds Ratios of the univariate and multivariate generalized linear models to assess the influence of the presence of RF, sex, type of delivery, healthcare service and birth weight on the occurrence of failure in the TEOAE screening

		Univariate Model			Multivariate Model					
					Main Effect			RF interaction		
		OR	CI 95%	p	OR	CI 95%	p	OR	CI 95%	p
**RE**	RF (Ref.: No)	4.49 ^††^	3.18–6.26	< 0.001*	2.82 ^†^	1.72–4.44	< 0.001*	–	–	–
	Sex (Ref.: Female)	1.84 ^†^	1.35–2.55	< 0.001*	2.03 ^†^	1.43–2.95	< 0.001*	1.69 ^†^	0.83–3.56	0.156
	Type of delivery (Ref.: vaginal)	1.20	0.87–1.67	0.277	0.95	0.66–1.40	0.801	0.73	0.35–1.57	0.408
	Health Service (Ref.: public)	1.19	0.87–1.64	0.277	1.56	1.11–2.19	0.010*	1.72 ^†^	0.88–3.38	0.116
	Weight (100g)	0.94	0.92–0.96	< 0.001*	0.97	0.95–1.00	0.051	0.94	0.90–0.99	0.030*
**LE**	RF (Ref.: No)	2.78 ^†^	2.02–3.78	< 0.001*	2.03 ^†^	1.26–3.15	0.005*	–	–	–
	Sex (Ref.: Female)	1.93 ^†^	1.46–2.58	< 0.001*	2.14 ^†^	1.52–3.07	< 0.001*	1.48	0.75–3.04	0.267
	Type of delivery (Ref.: vaginal)	1.16	0.87–1.56	0.312	0.90	0.63–1.28	0.542	0.58	0.29–1.19	0.138
	Health Service (Ref.: public)	1.23	0.93–1.60	0.140	1.35	0.98–1.87	0.070	1.39	0.72–2.66	0.320
	Weight (100g)	0.96	0.94–0.98	< 0.001*	0.97	0.95–1.00	0.028*	0.94	0.90–0.99	0.013*

Abbreviations: CI, Confidence Interval; LE, left ear; OR, Odds Ratio; RE, right ear; RF, risk factors. *: statistically significant value at the 5% level (p ≤ 0.05); †: small effect; ††: medium effect.

The analysis showed that for the right ear, sex and healthcare type significantly impacted TEOAE failure odds. Males had 2.03 times higher odds, and private healthcare increased odds by 1.56 times. For the left ear, males had 2.14 times higher odds of failure.

Table 6 presents the post hoc analysis conducted to explain the interactions identified by the established model.

**Table 6:** Post hoc analyses regarding the interactions of risk factors with sex, healthcare service and birth weight for the right ear and the interaction of risk factors with birth weight for the left ear

	Effect of the presence of RF based on sex, healthcare service and birth weight.					Effect of sex, healthcare service and birth weight based on the presence of RF				
			OR	CI 95%	P		RF	OR	CI 95%	P
RE	Sex	Fem.	2.17 ^†^	1.09–4.30	0.054	Sex (Ref.: Fem.)	No	1.56	1.07–2.29	0.044*
		Male	3.67 ^††^	2.25–5.97	< 0.001*		Yes	2.64 ^†^	1.43–4.89	0.004*
	Healthcare Service	Pub.	2.15 ^†^	1.22–3.81	0.016*	Healthcare service (Ref.: Pub.)	No	1.19	0.82–1.74	0.734
		Priv.	3.70 ^††^	2.05–6.66	< 0.001*		Yes	2.04 ^†^	1.17–3.57	0.024*
	Weight	M–1 SD	3.86 ^††^	2.41–6.19	< 0.001*	Weight (100 g)	No	1.00	0.96–1.04	> 0.999
		M + 1 SD	2.06 ^†^	1.11–3.83	0.044*		Yes	0.95	0.92–0.98	0.002*
LE	Weight	M–1 SD	2.82 ^†^	1.82–4.36	< 0.001*	Weight (100 g)	No	1.00	0.97–1.04	> 0.999
		M + 1 SD	1.47	0.80–2.68	0.426		Yes	0.92	0.92–0.98	0.002*

Abbreviations: CI, Confidence Interval; LE, left ear; OR, Odds Ratio; RE, right ear; RF, risk factors; *: statistically significant value at the 5% level (p ≤ 0.05); †: small effect; ††: medium effect.

Table 6 shows that for the right ear, individuals with RF had higher odds of TEOAE failure, significantly so for those with lower birth weight. For the left ear, RF also increased failure odds, but only among those with lower birth weight.

Table 7 presents the analysis of the influence of RF, sex, delivery type, healthcare service, and birth weight on aABR failures using univariate and multivariate logistic GzLM for each ear.

**Table 7:** Odds Ratios from univariate and multivariate generalized linear models to assess the influence of the presence of RF, sex, type of delivery, healthcare service, and birth weight on the occurrence of failure in the aABR

			Univariate Model			Multivariate Model	
		OR	CI 95%	p	OR	CI 95%	p
RE	Sex (Ref.: Female)	1.77 ^†^	0.98–3.34	0.067	1.85 ^†^	1.01–3.51	0.052
	Type of delivery (Ref.: vaginal)	1.35	0.70–2.82	0.388	1.08	0.55–2.29	0.825
	Health Service (Ref.: public)	2.20 ^†^	1.23–3.95	0.008*	2.21 ^†^	1.22–4.02	0.009*
	Weight (100g)	0.97	0.94–1.01	0.090	0.97	0.93–1.00	0.047*
LE	Sex (Ref.: Female)	1.84 ^†^	0.98–3.61	0.066	1.95 ^†^	1.03–3.85	0.046*
	Type of delivery (Ref.: vaginal)	1.17	0.60–2.45	0.666	0.96	0.48–2.05	0.910
	Health Service (Ref.: public)	1.76 ^†^	0.96–3.25	0.066	1.80 ^†^	0.97–3.36	0.062
	Weight (100g)	0.97	0.93–1.00	0.069	0.96	0.93–1.00	0.035*

Abbreviations: CI, Confidence Interval; LE, left ear; OR, Odds Ratio; RE, right ear; RF, risk factors; *: statistically significant value at the 5% level (p ≤ 0.05); †: small effect; ††: medium effect.

The analysis showed that healthcare type significantly affected aABR failure odds only in the right ear, with private/supplementary healthcare having 2.21 times higher odds than public healthcare. Sex significantly impacted failure odds in the left ear, with males having 1.95 times higher odds. Birth weight also affected failure odds in both ears, with a 100g decrease in birth weight increasing failure odds by 1.03 times in the right ear and 1.04 times in the left ear.

## Discussion

Implementing and maintaining neonatal hearing screening services is a significant challenge in developing countries, 6 especially in meeting the goals and quality indicators outlined in the guidelines. 7 8 Universal screening for all live newborns is recommended, with a target coverage rate of 95%, aiming for 100%. 7 8 In this service, the coverage rate reached 99.96%, comparable to developed countries' programs. This high coverage is attributed to the daily presence of audiologists and efficient management, with expected losses, deaths, and transfers.

Although Federal Law 12,303 of 2010 mandates neonatal hearing screening, 16 Brazil's coverage rate and procedures still fall short of expectations. Despite an increase in coverage over the years, significant regional disparities persist. An ecological study showed a national increase in coverage, 9 but the overall rate remains low (37.2%), far below the recommended rate, highlighting regional inequalities. 9 In Sergipe, 17 the coverage rate was under 40%, and unlike other regions, it has not improved over time. A review revealed that only the South and Southeast regions reached a 95% coverage rate. 18

The selection protocol for procedures follows established guidelines. 7 8 In this service, TEOAE is used for newborns without risk factors, while both TEOAE and aABR are used for those with risk factors. It was found that 88.88% of newborns had no risk factors, while 11.12% had some, aligning with the Ministry of Health's estimate that about 10% of newborns have risk factors. 8 The literature on the choice of procedures for hearing screening varies, both nationally 18 and internationally. 10

Figure 2 shows the neonatal hearing screening results for newborns without risk factors. Of the 7,846 newborns, 97.51% passed the TEOAE, and 1.19% were referred for retesting after discharge. These results are similar to a Brazilian study where the "pass" rate ranged from 95.70% to 98.00% in 2016-2017. 19 In a previous study at the same hospital, 96.86% passed, and 3.12% failed the TEOAE. 20 Of the 195 who failed, 131 underwent aABR before discharge, and 102 (77.86%) passed, supporting the combined methodology to reduce follow-up referrals and non-attendance. 7 8

Figure 4 shows the neonatal hearing screening results for newborns with risk factors. Of the 982 newborns, 94.09% passed the aABR and were referred for audiological follow-up, while 5.91% were referred for audiological diagnosis. These results are similar to another Brazilian study, where 93% passed and 7% failed, 19 and to our previous study, where 95.64% passed and 4.36% failed. 20

For follow-up of newborns without risk factors for retesting after discharge, the evasion rate was 8.60%, while for newborns with risk factors referred for audiological diagnosis, it was 8.62%. Both rates were similar, suggesting that even though newborns with risk factors may have more compromised health, newborns without risk factors also fail to return for services. In developing countries like Brazil, factors such as lack of money, difficulty accessing follow-up locations, and lack of understanding of the importance of retesting/diagnosis may explain these evasions. 21 22

Despite evasion being a challenge for neonatal hearing screening services, attendance for retesting/diagnosis is a key quality indicator. The evasion rates found in this study were lower than those reported in a literature review, where retesting evasion ranged from 9% to 34% in mixed private hospitals, and diagnostic evasion ranged from 28% to 100%. To reduce evasion, strategies such as implementing search and monitoring systems, humanized care, and developing educational materials for families are crucial. 23

The percentage of referrals for diagnosis in this study was 0.71%, similar to another Brazilian study (0.7%). 24 This rate is below the guidelines' recommendation that 2% to 4% of newborns should be referred for diagnosis. 7 8 Figures 3 and 5 present diagnosed cases of hearing loss for both newborns with and without risk factors. According to the World Health Organization, the average prevalence of hearing loss in the neonatal period is 2/1000. 6 A meta-analysis showed a prevalence of 1.1 per 1,000 screened newborns, which was 6.9 times higher among NICU admissions. 25

A Brazilian study with 11,900 newborns screened at birth found the overall occurrence of permanent congenital hearing loss to be 2/1000 live births, while for newborns with risk indicators, it was 18.6/1000. 26 In this study, the prevalence of hearing loss in newborns without risk factors was 0.63/1000 (0.06%), while for those with risk factors, it was 11.2/1000 (1.12%). The prevalence of hearing loss in newborns without risk factors was lower than most literature reports, 7 8 while the prevalence in newborns with risk factors was similar to previous studies.

The lack of systematization in data collection and analysis makes it difficult to determine the true prevalence of hearing loss in newborns, both in Brazil and globally. 27 Results on hearing loss prevalence should be interpreted cautiously due to the lack of methodological consistency in screening processes and high dropout rates during retesting or diagnostic stages. 18

Although this service uses aABR and TEOAE procedures for neonatal hearing screening in newborns with risk factors, no cases of Auditory Neuropathy Spectrum Disorder (ANSD) were diagnosed. The literature shows discrepancies in ANSD prevalence, ranging from 0.5% to 15%. 28

Table 2 shows the failure rates in the TEOAE and aABR tests by ear. For TEOAE, the left ear (2.48%) had a higher failure rate than the right ear (1.95%). For aABR, the right ear (4.99%) had a higher failure rate than the left ear (4.49%). However, none of these differences were statistically significant. The literature suggests that the left ear often shows a higher failure rate in the TEOAE test, 19 29 while no significant differences are found between ears in the aABR test. 30

The results from Tables 5 and 7 show that, despite using different procedures (TEOAE and aABR), the private/supplementary healthcare service had a 1.56-fold higher odds of failure in TEOAE and 2.21-fold higher odds in aABR compared with the public healthcare service. This highlights the importance of strengthening Brazil's Unified Health System (SUS), as the number of individuals with private health insurance has slightly increased, while the number of screened newborns has significantly risen nationwide. 9

Minimizing delays in early diagnosis of hearing loss is crucial for ensuring that all children have auditory access. 31 It can support public healthcare and emphasize the importance of existing public policies, despite the ongoing challenges in the national neonatal screening program. 32

In early detection of hearing loss, Primary Health Care (PHC) plays a crucial role. PHC, through the Family Health Strategy (FHS), aims to shift away from the traditional disease-focused care model and has been recognized globally as an effective approach to improving healthcare access and population health. 33 However, despite the inclusion of speech-language pathologists in the SUS, their role is mainly concentrated in high-complexity settings, such as university hospitals and maternity wards. 34

According to statistical analysis ( Tables 5 , 6 , and 7 ), the likelihood of failure in both TEOAE and aABR procedures was higher among male newborns. Males had a 2.03 times higher chance of failure in the right ear and a 2.14 times higher chance in the left ear compared to females. A Brazilian study showed no significant difference, but observed that more males failed (10.4%) compared to females (8.1%). 35 Another study found that male newborns were 1.5 times more likely to fail, 36 suggesting that females may have greater amplitude in frequency bands during the TEOAE test. 37

Table 7 revealed that birth weight significantly affectd the likelihood of failure in the aABR test. Lower birth weight increases the chances of failure in both ears. This aligns with existing literature, which shows that premature neonates with very low birth weight have higher failure rates in hearing screening and a higher occurrence of auditory impairments. 38 Additionally, birth weight under 1500 g notably increases the chances of failure in neonatal hearing screening. 39

The findings offer valuable insights for the enhancement of public policies in neonatal hearing health but should be interpreted within the context of the specific setting studied. Conducted in a single healthcare center, the study's results may not be generalizable to other regions of the country. Additionally, the non-randomized sample could introduce bias, and the lack of control over variables like genetic or environmental factors presents a limitation.

## Conclusion

This study described the results of one year of a UNHS service, emphasizing the importance of such programs for early identification and intervention, especially in developing countries like Brazil. The coverage rate was 99.96%, with 63 newborns (0.71%) referred for diagnosis and 16 (0.18%) confirmed with hearing loss. A significant interaction between sex and healthcare service type was found for TEOAE failures, while birth weight impacted the likelihood of failure in aABR.

Future research should explore the long-term outcomes of early intervention, the effectiveness of risk-based screening, and strategies to overcome implementation challenges.
